# Mesenchymal Stem Cell-Derived Exosomes: Emerging as a Promising Cell-Free Therapeutic Strategy for Autoimmune Hepatitis

**DOI:** 10.3390/biom14111353

**Published:** 2024-10-24

**Authors:** Liwen Wu, Longze Zhang, Minglei Huang, Yan Wu, Sikan Jin, Yaqi Zhang, Xinyun Gan, Ting Yu, Guang Yu, Jidong Zhang, Xianyao Wang

**Affiliations:** 1 Department of Immunology, Zunyi Medical University, Zunyi 563003, China; 2 Collaborative Innovation Center of Tissue Damage Repair and Regeneration Medicine, Zunyi Medical University, Zunyi 563000, China; 3 Scientific Research Center, The Third Affiliated Hospital of Zunyi Medical University, Zunyi 563003, China

**Keywords:** mesenchymal stem cells, cell-free therapy, exosomes, autoimmune hepatitis, targeted delivery, immunomodulation

## Abstract

Autoimmune hepatitis (AIH) is an immune-mediated liver disease that currently faces limited treatment options. In its advanced stages, AIH can progress to liver fibrosis and cirrhosis. Recent research has increasingly focused on cell-free therapies, particularly the use of mesenchymal stem cell (MSC)-derived exosomes (Exos), which have shown promise in treating autoimmune diseases, including AIH. MSC-Exos, as microvesicles with low immunogenicity, high safety, and permeability, can deliver RNA, DNA, proteins, lipids, and various drugs for disease treatment, showing promising clinical application prospects. This review provides a comprehensive summary of the current research on MSC-Exos in the treatment of autoimmune hepatitis (AIH) and explores the underlying molecular mechanisms involved. It highlights the significant regulatory effects of MSC-Exos on immune cells and their ability to modify the microenvironment, demonstrating anti-inflammatory and anti-fibrotic properties while promoting liver regeneration. Additionally, this review also discusses potential challenges and future strategies for advancing Exo-based therapies in the treatment of AIH.

## 1. Introduction

**Figure 1:**
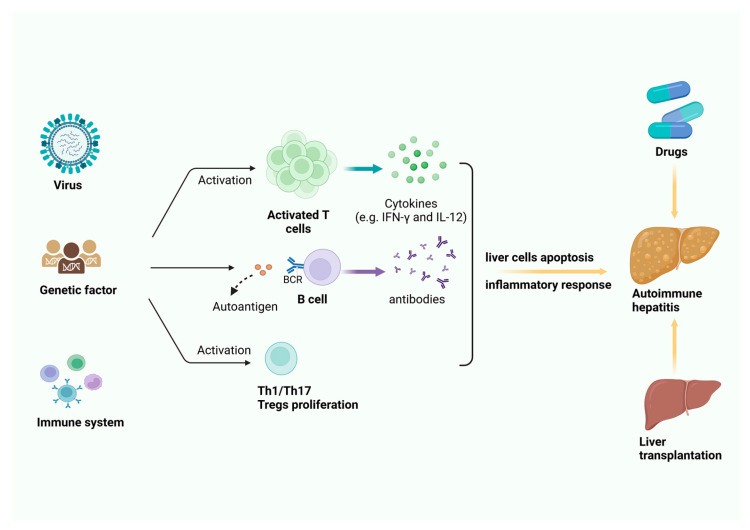
The mechanisms and current primary treatment strategies by drugs and liver transplantation in later periods with liver cirrhosis for AIH.

Autoimmune diseases (AID) are conditions in which an imbalance in the immune system leads to the attack on cells, tissues, and organs, resulting in damage. Common AIDs include systemic lupus erythematosus, rheumatoid arthritis, type 1 diabetes, and autoimmune hepatitis (AIH) [1,2]. AIH is a liver disease resulting from an immune homeostasis abnormality, marked by chronic or progressive damage to liver cells. In recent years, the incidence of autoimmune diseases (AIDs) has been steadily increasing [3]. In Asia, the incidence of AIH is approximately one to two cases per 100,000 people, with women being more commonly affected, at a male-to-female ratio of about 1:4. While AIH can occur at any age, the peak incidence is typically observed between 14 and 60 years old [4,5]. The pathogenesis of AIH remains unclear, but current research suggests that factors such as immune dysfunction, genetics, and viral infections contribute to Treg cell apoptosis, imbalances in T cell subpopulations, increased secretion of B cell cytokines, and the promoted production of autoantibodies, all of which are key contributors to the development of AIH. Additionally, this process is accompanied by an increase in various pro-inflammatory cytokines, including TNF-α, IFN-γ, IL-1, IL-6, IL-12, and IL-17 [6,7,8,9]. In the early stages, AIH patients may experience symptoms such as fatigue, itching, epigastric discomfort, and loss of appetite [10]. As the disease progresses to late-stage liver cirrhosis, clinical manifestations primarily include jaundice, spider nevi, hepatosplenomegaly, ascites, and splenomegaly [11]. A significant increase in serum transaminases and immunoglobulin G is a key feature for diagnosing AIH. Additionally, AIH patients often have various non-specific auto-antibodies in their serum, such as anti-nuclear antibodies, anti-smooth muscle antibodies, and anti-liver-kidney microsomal antibodies [12,13]. Based on the type of auto-antibodies present, AIH can be classified into type 1 and type 2, with type 1 being more common. Type 1 AIH typically involves the presence of anti-nuclear antibodies and/or anti-smooth muscle antibodies, while type 2 AIH is characterized by the high expression of anti-liver-kidney microsomal-1 and/or anti-liver-kidney microsomal-3, and/or anti-liver cytosol-1 antibodies [14,15]. Currently, the treatment of early-stage AIH primarily includes immunosuppressants such as prednisone, azathioprine, and budesonide, as well as second-line medications like mycophenolate mofetil [16,17]. When hepatitis progresses to advanced liver fibrosis or cirrhosis, liver transplantation may be considered (Figure 1). However, the current main treatment approaches for AIH still face a range of challenges. Recent studies indicate that approximately 20% of AIH patients experience intolerance and diminished quality of life following drug therapy, with some encountering complex issues such as inconsistent efficacy [18,19]. Additionally, due to the shortage of donors and the presence of immune rejection following transplantation, liver transplants for late-stage AIH patients remain significantly limited. Therefore, there is an urgent need for new therapies with fewer side effects and better efficacy to more effectively control the progression of AIH and improve treatment outcomes.

MSCs are a type of stem cell with robust self-renewal capacity, anti-inflammatory properties, and tissue repair capabilities. Recent research has indicated that MSC therapy may be a promising strategy for treating AIH, as targeted MSC injections have been shown to effectively reduce liver inflammation in AIH mouse models [20,21,22]. MSCs can also secrete cell vesicles containing various biologically active molecules, known as exosomes (Exo). MSC-Exos have immunomodulatory properties similar to those of MSCs, such as inhibiting the proliferation of T and B cells, modulating macrophage differentiation, and suppressing NK cell cytotoxicity, making them a feasible option for AIH treatment [23,24]. As a cell-free therapy, MSC-Exos offer unique advantages in AIH treatment, including lower immunogenicity and better safety and permeability compared to MSCs [25,26]. This study reviews the current research on MSC-exosome therapy for AIH, explores the potential mechanisms involved in promoting tissue repair and regulating the immune system, and discusses possible applications in AIH treatment.

## 2. Methods

### 2.1. Search Strategy

We conducted a search in three databases (Pubmed, EMBASE, and Web of Science) for relevant English studies that met the criteria for this analysis up to September 2024. During the search process, the following search terms were utilized: “Autoimmune Hepatitis, Mesenchymal stem cells, Exosomes and mesenchymal stem cell (MSC)-derived exosomes”.

### 2.2. Eligibility Criteria

Studies meeting the following criteria will be included: 1. English-language research; 2. studies reporting on AIH patients treated with MSC-Exo therapy; 3. clinical research, cell studies, animal experiments, reviews, and meta-analyses.

## 3. MSCs and Exos

MSCs were initially discovered in bone marrow. In recent years, with rapid advancements in scientific research, MSCs have also been detected in various tissues, such as the umbilical cord, amniotic fluid, adipose tissue, placenta, endometrium, and dental pulp [27]. Despite differences in tissue origin, MSCs consistently express CD73, CD105, and CD90 while lacking CD45, CD34, CD14, CD11b, CD19, and human leukocyte antigen-II [28]. MSCs are characterized by self-renewal, immune modulation, and tissue and organ regeneration capabilities [29]. Under various stimulating factors, MSCs can differentiate into adipocytes, osteocytes, chondrocytes, and hepatocytes [30,31,32]. Additionally, MSCs exhibit chemotactic migration properties, enabling them to move directionally to sites of injury or inflammation. Furthermore, MSCs have immunomodulatory potential, including the ability to suppress the proliferation, activation, and differentiation of various immune cells such as natural killer cells, dendritic cells, macrophages, B cells, and T cells [33,34,35]. These properties make MSCs applicable in treating conditions such as cancer, liver fibrosis, liver failure, AIDs, and myocardial ischemia [36,37].

**Figure 2:**
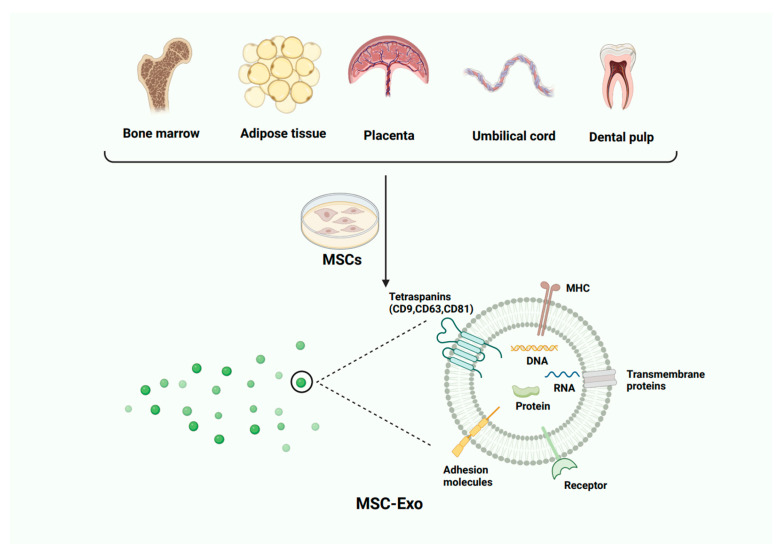
MSCs are derived from various sources and the Exos secreted by MSCs can encapsulate various substances, including DNA, RNA, and proteins.

Exos are small secretory vesicles with a lipid bilayer membrane, ranging from 30 to 100 nm in diameter, containing a diverse array of molecules such as proteins, DNA, lipids, mRNAs, and various non-coding RNAs [38,39,40]. These components not only stabilize the biological activity of Exos and enhance their targeting capabilities but also regulate gene expression in recipient cells. Exos express a variety of proteins, such as heat shock proteins, lipid-associated proteins, phospholipases, membrane transport proteins, and fusion proteins [41,42]. Surface membrane proteins are commonly recognized as non-specific markers of exosomes, including CD81, CD9, and CD63, as well as MSC surface markers CD73 and CD90 (Figure 2). Additionally, membrane proteins facilitate the transport of Exos and promote their fusion with target cells either directly or through receptor-ligand pathways, thereby enabling the Exos to exert their biological effects within the cells [43]. The lipid membrane encapsulating Exos could protect the contents from the external environment, thereby preserving its biological activity. Upon entering target cells, Exos release their contents, which influence intracellular signaling pathways and modulate cell activity and function. Notably, the RNA within Exos plays a crucial role in this process [44,45]. The RNA includes mRNAs, microRNAs, and ncRNAs. Among these, miRNAs are of particular interest due to their pivotal role in biological processes. To better understand the miRNA species contained in MSC-Exos, according to https://guolab.wchscu.cn/EVmiRNA/ accessed on 10 October 2024, the significantly expressed miRNAs mainly include miR-4792, miR-1246, miR-6089, etc., and there are also some specific miRNAs, such as miR3665 and miR658. Although it is now clear that for exosomes from different cellular sources, there are differences in the types of miRNAs present, there is no clarity as to whether there are differences for MSCs from different preconditioning pathways as well as different tissue sources. It has been reported that MSC-Exos retain most of the functions of their source MSCs, including immune regulation, anti-inflammatory, and anti-fibrotic effects, as well as promoting tissue growth [46]. Research also indicates that MSC-Exos, depending on their tissue source and pretreatment, exhibit distinct characteristics and biological effects. For example, MSC-Exos induced under hypoxic conditions can promote angiogenesis through vascular endothelial growth factor [47]. The primary function of bone marrow MSC-Exos lies in promoting tissue regeneration, while Exos derived from adipose MSCs are more focused on immunomodulatory effects [48].

Although available research indicates that MSCs hold significant potential for clinical applications, direct injection of MSCs into target organs or tissues is often hampered by immune rejection, which greatly reduces the number of MSCs that reach the specific site and function effectively, thus limiting therapeutic efficacy. Additionally, the potential tumorigenic risk of MSCs should not be overlooked [49,50]. In comparison, MSC-Exos provide better targeting capabilities owing to their smaller size, which enables them to navigate various physiological barriers effectively [51,52]. Furthermore, MSC-Exos have lower immunogenicity, reducing clearance by immune cells and allowing for a longer duration of action in vivo [53]. Therefore, MSC-Exos, as an acellular therapy, avoid the negative effects associated with MSCs, making them a better option for clinical application.

## 4. MSC-Exos Treatment in AIH

MSC-Exos have been shown to be effective in treating AIH, primarily due to their function in delivering microRNAs (miRNA). Early studies on MSC-Exos in AIH demonstrated that Exos derived from bone marrow MSCs infected by pre-miR-223 were engineered to express high levels of miR-223. These Exos reduced the production of nucleotide-binding oligomerization domain-like receptor family pyrin domain-containing 3, thereby inhibiting hepatic inflammation and cell death, ultimately providing liver protection [54]. The miR-223-3p within MSC-Exos exhibited similar effects. MSC-Exos, enriched with miR-223-3p through pretreatment, effectively delivered these miRNAs to target cells, reducing the release of inflammatory cytokines in the liver and macrophages. This was achieved by modulating miR-223-3p levels and regulating signal transducers and activators of transcription 3 (STAT3) expression in both liver cells and macrophages, consequently alleviating the inflammatory response in AIH [55]. Meanwhile, another study found that MSC-Exos can serve as drug carriers. Incorporating dexamethasone (DEX) into MSC-Exos significantly enhances the targeting efficiency of DEX and the combination of DEX with MSC-Exos exhibits a synergistic therapeutic effect, resulting in significantly improved efficacy compared to treatment with Exos or DEX alone [56]. Interestingly, other researchers have utilized nicotine to induce MSCs and collected conditioned media (CM). Treatment of AIH mice with CM led to significant reductions in the levels of TNF-α, IFN-γ, and IL-6, while increasing IL-4 anti-inflammatory gene expression [57]. Though this essay does not specify the functional ingredients or mechanisms of CM, it is well-known that Exos can be derived from CM. A recent study observed that in MSC-EV treatment of AIH, in which CD4 T cells are the primary target cells, MSC-EV reduced CD4 T cell activation by decreasing glycolysis, enhancing mitochondrial oxidative phosphorylation, and thereby inhibiting the release of cytokines (IFN-γ) compared to the control group. This effect may be attributed to the abundance of mitochondrial proteins in MSC-EV [58]. Therefore, MSC-Exos may play a significant role in this process [Table 1].

Currently, many studies focus on elucidating the mechanisms and efficacy of MSC-Exos in treating hepatitis, cirrhosis, liver fibrosis, and liver failure. A pooled analysis based on the treatment of liver disease with MSC-EV demonstrated that the efficacy of multiple sample treatments, different sources, different routes of injection, and different numbers of treatments was evaluated. The findings indicated that this alteration was predominantly observed in AST and ALT levels, suggesting that MSC-EV has considerable potential for both acute and chronic liver disease [59]. In order to gain a more comprehensive understanding of MSC-based studies in AIH clinical trials, a search was conducted at Clinicaltrials.gov for relevant trials. It was found that, at present, only studies based on MSC injection therapy to alleviate the symptoms of AIH are available. However, for the complications associated with advanced AIH, such as liver fibrosis, cirrhosis, and liver failure, MSC-Exo-related clinical studies have been conducted (Table 2). Therefore, further research should be conducted to explore the efficacy, safety, therapeutic dosage, and in vivo accumulation of MSC-Exos in the clinical treatment of AIH.

## 5. Exo Modification Enhancing Liver Targeting

**Figure 4:**
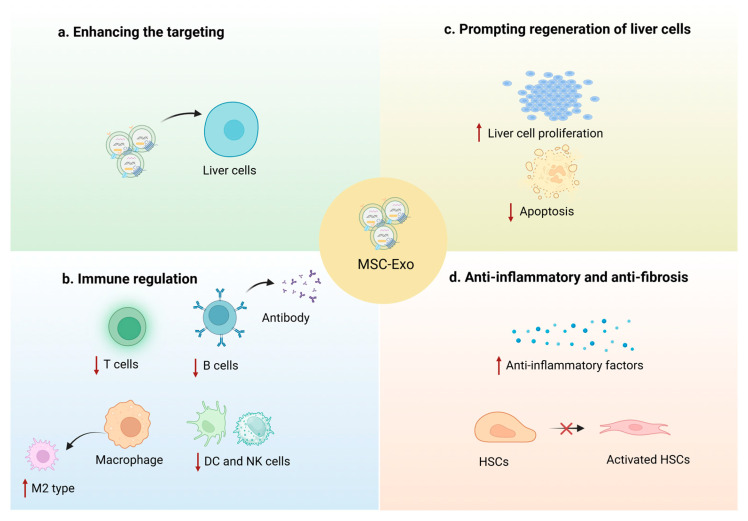
The underlying mechanism of MSC-Exos in AIH. MSC-Exos could deliver small molecules or drugs to targeted tissue. (**a**) Enhancing the targeting. Modification of the Exos or MSCs to gather targeting Exos and enhance the targeting ability to issue [56,71,72]. (**b**) Exo regulating immune function. Exos can reduce B cell activation and the production of antibodies [79], inhibit the ability of T cells [88,89,90], promote macrophage polarization to anti-inflammatory M2 type, and control DC and NK cell levels [95,103,105]. (**c**) MSC-Exos promoting liver cell regeneration. Exos deliver cytokines to promote liver cell regeneration and inhibit apoptosis [108,110]. (**d**) MSC-Exo anti-inflammatory and anti-fibrosis. Exos mainly promote the level of anti-inflammatory factors and regulate the activation of HSCs to anti-inflammatory and anti-fibrosis [116,120].

Similar to MSCs, MSC-Exos also possess the ability to migrate to sites of injury and inflammation. However, due to the extensive blood flow in the liver, some Exos are phagocytized by macrophages or cleared by the kidneys, which reduces the number of MSC-Exos that ultimately reach the liver [60,61,62]. To address this issue, researchers have found that loading MSC-Exos with specific RNA or drugs can significantly enhance the therapeutic efficacy and increase the hepatic accumulation of the loaded substances. Additionally, physical, chemical, and genetic engineering modifications can add functional ligands and enhance the specific targeting ability of MSC-Exos (Figure 4a).

### 5.1. MSC-Exos Prompting Targeting of Therapeutics in Hepatic Diseases

Multiple studies have found that loading small molecules or drugs designed to treat hepatitis and liver fibrosis into MSC-Exos results in better therapeutic outcomes compared to treatment with either the drugs or MSC-Exos alone. It has been demonstrated that Exos can enhance enrichment efficiency in the liver by loading miR-455-3p, miR-223, miR-17, miR-181-5p, and circDIDO1, thereby reducing liver injury [63]. Zhao et al. [56] reported that Exos derived from MSCs loaded with DEX (Exo@DEX) injected into AIH model mice significantly improved Exo targeting, with Exo@DEX delivering DEX to the liver 2.6 times more effectively than free DEX. Additionally, another study showed that loading obeticholic acid (OCA) and luteolin (LUT) into MSC-Exos could significantly enhance the therapeutic efficiency in treating liver fibrosis [64,65]. Overall, these approaches have the potential to improve the efficiency of Exo delivery to specific tissue sites and enhance their targeting. However, further investigation is needed to determine the feasibility of using MSC-Exos loaded with a broader range of therapeutic agents to alleviate the symptoms of AIH.

### 5.2. MSC-Exos Modification Increasing Their Targeting Properties

Physical modifications, such as ultrasound, electroporation, filtration, or centrifugation, can concentrate and load more small molecules; however, these methods may disrupt the structure of MSC-Exos, limiting their functional efficacy [66]. Therefore, current research focuses on exploring the benefits of chemical and genetic modifications for improving the therapeutic efficiency of MSC-Exos. Chemical modifications often involve co-incubating MSC-Exos with targeting peptides or covalently conjugating ligands to the lipid or protein components of MSC-Exos using different linkers (e.g., targeting the transferrin receptor) [67]. Genetic modifications primarily involve modifying the source cells of MSC-Exos using plasmid vectors to alter the targeting properties of the Exos [68].

**Figure 3:**
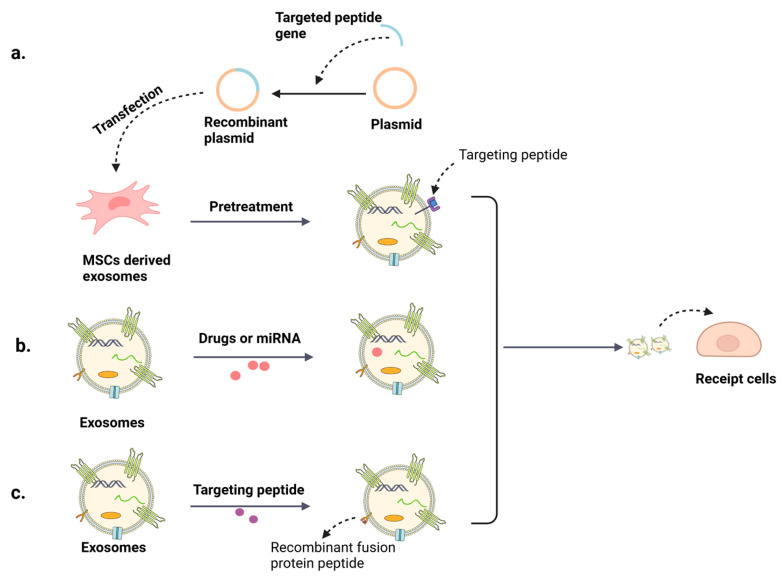
Strategies for Exo-targeted modification. (**a**) Building recombinant plasmid with targeted peptide genes and then using them to transfect the MSCs, so that they can produce the targeting Exos which carry the special targeting peptide [73]. (**b**) Exos loaded with drugs or miRNA [56,63]. (**c**) Binding of exosomal peptides to tissue-specific targeting peptides confers targeting of exosomal tissues [75].

It is well acknowledged that modified MSC-Exos can be redistributed to tissues such as the heart, brain, intestines, liver, lungs, and pancreas. Combining the cardiac-targeting peptide (CTP) with the exosomal peptide Lamp2b in a vector construction resulted in a 16% increase in modified MSC-Exo delivery efficiency compared to controls. Additionally, creating recombinant fusion proteins containing the RGD-4C peptide (ACDCRGDCFCG) or the CRPPR peptide is another method to enhance myocardial targeting [69,70]. Sarkar et al. [71] utilized genetic engineering techniques to generate CAP-Lamp2b fusion protein from a functional AAV coat-specific peptide (CAP) and Lamp2b peptide on the surface of MSC-Exo membranes, which resulted in a 20% increase in MSC-Exo translocation to the brain. Similarly, Han et al. [72] used the fusion gene *iRGD-Lamp2b* to modify MSC-Exos, observing a predominant enrichment in the colon. Based on the target cells of MSC-Exos and the targeting peptide HSTP1 in hepatic fibroblasts (HSCs), Lin et al. [73] constructed a fusion gene of Lamp2b and HSTP1 in a plasmid. They added a glycosylation motif GNSTM at the N-terminal end to prevent hydrolysis of the target peptide. This plasmid was then transfected into MSCs to prepare HSTP1-Exos. A series of experiments revealed that HSTP1-Exos were efficiently delivered to HSCs both in vivo and in vitro compared to the control group. In vivo experiments also showed that the HSTP1-Exos group experienced less endocytosis by macrophages and contained more MSC-Exos in the liver, significantly improving the targeting of Exos and thus attenuating HSCs deposition and resisting fibrosis. Other methods, such as using coppexoer-catalyzed azide-alkyne cycloaddition, targeted nanoantibodies/antibodies, and hydrophobic interactions/membrane engineering to shape MSC-Exo surfaces, can also enhance the targeting capabilities. [74]. Identifying peptides that are specifically and preferentially expressed by liver cells is expected to lead to the preparation of more liver-targeted MSC-Exos with high hepatic targeting efficiency. Moreover, studies have demonstrated that coupling the hepatocellular carcinoma-targeting peptide (SP94) with exosomal peptide CP05 can redistribute MSC-Exos [75]. Therefore, the aforementioned strategies represent potential approaches to enhance the targeting of MSC-Exos (Figure 3).

## 6. Underlying Mechanism of MSC-Exos in AIH

Increasing evidence suggests that MSC-Exos can enter cells via specific receptors and release encapsulated small molecules, thereby influencing the proliferation, activation, and function of immune cells by regulating various signaling pathways. Consequently, MSC-Exos exhibit significant therapeutic potential for AIH, which is characterized by increased auto-antibody production and T-cell dysfunction. To elucidate the regulatory abilities of MSC-Exos on immune cells, this section will focus on their immunomodulatory effects on B cells, T cells (especially CD4+ T cells), macrophages, and other immune cells, as well as their underlying mechanisms (Figure 4b).

### 6.1. B Cells

Auto-antibodies secreted by plasma cells are important hallmarks of AIH. B cells exhibit distinct roles in immune responses based on their activation state. Activated and mature B cells can promote the progression of AIH by producing large quantities of antibodies, such as IgE, IgM, and IgG [76,77,78]. Recent studies have gradually illuminated the mechanisms by which MSC-Exos regulate B cell proliferation and activation. A study demonstrated that miR-125b within MSC-Exos directly binds to and downregulates the expression of *PR domain zinc-finger protein 1*, resulting in a reduced proportion of plasma cells and a corresponding decrease in antibody secretion [79]. Zhao et al. [80] co-cultured Exos derived from human umbilical cord MSC with peripheral blood from SLE patients, observing increased B cell apoptosis and significant inhibition of B cell proliferation and activation. Mechanistically, this effect may be due to MSC-Exos suppressing the expression of miR-155 and its target *SHIP-1* gene in B cells, thereby regulating B cells proliferation and activation through the inhibition of the ERK signaling pathway. Another study found that after co-culturing with MSC-Exos, the proportion of B cells in peripheral blood decreased by 18.33%, with most MSC-Exos being internalized by B cells. The research team hypothesized that once internalized, MSC-Exos regulate gene expression in B cells, primarily by downregulating genes associated with B cell activation and promoting IgM production. This ultimately leads to the inhibition of B cell activation and IgM antibody production [81]. The above studies demonstrate that MSC-Exos can inhibit B cell proliferation and activation while reducing antibody levels. This indicates that MSC-Exos may play a pivotal role in alleviating AIH symptoms by modulating B cell activity and immune responses.

### 6.2. T Cells

T cells can be subdivided into several subpopulations, including cytotoxic T cells, helper T cells, memory T cells, and regulatory T cells, based on their surface markers and distinct functional roles [82]. T cells can influence immune responses through various mechanisms, including cytokine secretion, direct cytotoxicity, and modulation of B cell antibody production [83,84,85]. T cell imbalances are frequently observed in AIH, and current research indicates that MSC-Exos may regulate T cell proliferation, differentiation, and migration. Studies have demonstrated that MSC-Exos can upregulate PD-L1 expression either by directly delivering PD-L1 or by transporting cytokines that promote PD-L1 production, thereby inhibiting T cell activation and promoting the proliferation of regulatory T cells [86,87]. Additionally, MSC-Exos can enhance the differentiation of monocytes from peripheral blood into regulatory T cells by increasing TGF-β levels, which attenuates excessive immune responses [88]. Studies have also found that MSC-Exos can inhibit T cell chemotaxis to inflammatory sites. MSC-Exos can deliver miR-223, which binds to the target gene *ICAM-1* (Intercellular Adhesion Molecule 1) and inhibits its expression, thereby reducing Th1 cell migration and adhesion and alleviating inflammation [89]. C-C motif chemokine ligand 21 (CCL21) recruits both resting and activated T cells, while CCL2 chemotactically recruits monocytes. It has been reported that MSC-Exos can suppress T cell migration by downregulating the expression of CCL21 and CCL2 [90]. Moreover, it has also been demonstrated that MSC-Exos can impede the proliferation of activated T cells by inducing cell cycle arrest [91]. Furthermore, research indicates that MSC-Exos can influence the balance between T helper 1 (Th1 cell), T helper 2 (Th2 cell), Th17 cells, and regulatory T cells (Treg cells). Chen et al. [92] demonstrated that MSC-Exos promote the transition from Th1 to Th2, which depends on a decrease in pro-inflammatory factors (e.g., IL-1β and TNF-α) and an increase in anti-inflammatory factors (e.g., TGF-β and IL-10). Mechanistically, MSC-Exo-derived sphingosine 1-phosphate reduces the Th17/Treg ratio by mediating Treg cell differentiation via S1P receptors on CD4+ T cells [93]. This underscores the significant regulatory impact of MSC-Exos on T cells by inhibiting pro-inflammatory effects.

### 6.3. Macrophages

Macrophages are a crucial cell type within the immune system and exhibit distinct subpopulation properties. Macrophages are primarily classified into M1 macrophages, which are pro-inflammatory and bactericidal, and M2 macrophages, which are anti-inflammatory, reparative, and immunomodulatory [94,95]. The transition between M1 and M2 macrophages is complex and influenced by various factors. Toll-like receptors (TLRs) have been shown to play a role in the regulation of macrophage polarization. Zhao et al. [96] found that MSC-Exos loaded with miR-182 could inhibit the expression of the TLR4 gene, thereby altering the polarization state of macrophages in a mouse model of myocardial ischemia/reperfusion injury. Additionally, research has demonstrated that MSC-Exos rich in miR-16 and miR-21 can promote macrophage polarization by regulating the Programmed Cell Death 4 and Phosphatase and Tensin Homolog pathways [97]. MSC-Exos can also deliver IL-10 to Kupffer cells and induce the expression of protein tyrosine phosphatase non-receptor 22, thereby promoting the transition of Kupffer cells to an anti-inflammatory phenotype and alleviating liver inflammation [98]. Furthermore, mesenchymal stem cells-derived extracellular vesicles (MSC-sEVs) can regulate M2 macrophage differentiation by upregulating TGF-β secretion, accelerating microvascular stabilization and functional recovery in severe spinal cord injury models [99]. Other studies have shown that in spinal cord injury models, macrophages that phagocytose BMSC-Exos can upregulate the expression of macrophage receptors with collagenous structure, thereby restoring normal physiological functions of macrophages, enhancing phagocytic activity in the injury site, and providing a new environment for tissue repair [100].

### 6.4. The Other Immune Cells

In addition to the immune cells previously mentioned, MSC-Exos can also modulate other immune cell types, including dendritic cells (DCs) and natural killer (NK) cells. DCs are considered to be the most functional antigen-presenting cells, and immature DCs have a strong migratory capacity, whereas activated DCs can effectively activate the initial T-cells and modulate the immune response in vivo [101,102]. He et al. [103] demonstrated that MSC-Exos with elevated indoleamine 2,3-dioxygenase 1 expression can modulate DC activity by downregulating markers such as CD40, CD86, CD80, and MHC-II while upregulating CD274 expression. These effects may also be associated with the presence of miR-540-3p carried by the MSC-Exo. NK cells primarily exert cytotoxic effects and are involved in immune surveillance and the development of AIDs [104]. Research indicates that MSC-Exos primarily inhibit the proliferation and activation of NK cells. Human fetal liver MSCs-Exo containing latency-associated peptide (LAP), TGF-β and thrombospondin 1 (TSP1) inhibit NK cell proliferation, activation, and cytotoxicity through the TGF-β/SMAD 2/3 (recombinant mothers against decapentaplegic homolog, SMAD) signaling pathway [105]. Therefore, MSC-Exos can influence various functions of immune cells through multiple mechanisms. Consequently, leveraging MSC-Exos to modulate the immune microenvironment and mitigate AIH symptoms could be a promising approach.

## 7. Prompting Regeneration of Liver Cells

In the progression of AIH, immune cells incorrectly target and damage liver cells, resulting in varying degrees of hepatic injury in affected individuals. However, recent studies have highlighted the potential of MSC-Exos in promoting liver regeneration and inhibiting hepatocyte apoptosis (Figure 4c). Research indicates that MSC-Exos can improve symptoms of acute liver injury by promoting hepatocyte proliferation, potentially through the translocation of neutral ceramidase and sphingosine kinase 2 to the regeneration site [106]. Additionally, Wang et al. [107] injected MSC-Exos into a mouse model of liver injury and ischemia-reperfusion injury, observing elevated levels of pro-regenerative factors such as Ki-67 antigen, proliferating cell nuclear antigen, and vascular endothelial growth factor, while levels of anti-regenerative factors, such as suppressors of cytokine signaling 3 and TGF-β, decreased. These changes collectively promoted liver regeneration and alleviated the disease. Furthermore, MSC-Exos can promote liver cell proliferation by delivering cytokines such as hepatocyte growth factor and fibroblast growth factor. This effect may be attributed to MSC-Exos reversing the suppressive impact of the inflammatory environment on regenerative factors, such as Wnt 2, β-catenin, and Cyclin D1 [108,109]. On the other hand, researchers have pointed out that in hepatic ischemia-reperfusion injury models, MSC-Exos dramatically decrease the level of apoptosis-driving complexes such as Caspase-8 and Caspase-9 through exogenous and endogenous apoptotic pathways, and ultimately inhibit the apoptosis of liver cells [110]. Oxidative stress is widely recognized as a key factor in promoting cell apoptosis. Prolonged oxidative stress disrupts the regulation of apoptosis inhibitors, thereby inducing cell death. However, MSC-Exos have been shown to mitigate this effect [111,112,113]. Studies have demonstrated that MSC-Exos can attenuate hepatocyte apoptosis by regulating oxidative stress in hepatocytes, primarily through the modulation of miRNAs in MSC-Exos that target *Phosphoinositide-3-Kinase Regulatory Subunit 1*, thereby activating the Phosphoinositide 3-Kinase/Protein Kinase B signaling pathway [114]. Meanwhile, Kong et al. [115] discovered that MSC-Exos effectively downregulate levels of oxidative damage markers, including malondialdehyde, cytochrome p450 2E1, and reactive oxygen species, in non-alcoholic steatohepatitis. Additionally, these MSC-Exos enhanced the activities of antioxidant enzymes, such as superoxide dismutase and glutathione, in hepatocytes, and elevated the p-Nrf2/Nrf2 ratio and the protein expression of NAD(P)H Quinone Dehydrogenase 1, which together help to suppress oxidative stress.

## 8. Anti-Inflammatory and Anti-Fibrosis

AIH is notably characterized by liver inflammation and carries the risk of developing into liver fibrosis. Previous studies have shown that MSC-Exos can ameliorate hepatitis caused by AIH and inhibit the fibrotic process (Figure 4d). The anti-inflammatory effects of MSC-Exos are largely attributed to the modulation of immune function. Additionally, MSC-Exos can deliver anti-inflammatory cytokines, such as IL-10 and TGF-β, to target cells and enhance the levels of anti-inflammatory factors [116]. Furthermore, MSC-Exos can modulate inflammatory signaling pathways through the delivery of miRNA, thereby alleviating inflammation. For example, Jiang et al. [117] found that miR-145-5p carried by MSC-Exos targets TLR4 and regulates the TLR4/nuclear factor kappa-light-chain-enhancer of activated B cells signaling pathway, thus modulating the progression of inflammation.

In this portion, we mainly discuss how the anti-fibrotic mechanism of MSC-Exos is related to the activation of HSCs and the change in the liver tissue microenvironment. During the progression of liver fibrosis, HSCs can proliferate rapidly and secrete large amounts of extracellular matrix [118,119]. Thus, targeting and inhibiting HSC activity is a key strategy for mitigating the symptoms of liver fibrosis. Current research has confirmed that MSC-Exos can inhibit HSCs proliferation by delivering small molecules that modulate the signaling pathways activated in HSCs. Li et al. [120] discovered that MSC-Exos could inhibit the proliferation of HSCs by downregulating the expression of protein kinase R-like endoplasmic reticulum kinase and apoptosis-promoting transcription factor C/EBP homologous protein in HSC-cholangioid organs and hepatocyte-cholangioid organs. Concurrently, Wang et al. [121] demonstrated that MSC-Exos can deliver miR-3-3p to inhibit the expression of TGFβRII, leading to the inactivation of SMAD signaling. Consequently, HSC activation is hindered, and the progression of liver fibrosis is slowed. Moreover, some miRs expressed in MSC-Exos have been proven to have anti-fibrotic capabilities. For instance, miR-378c has been shown to inhibit epithelial-mesenchymal transition by targeting the S-phase kinase-associated protein 2, thereby suppressing the activation of HSCs [122]. Additionally, MSC-Exos have been shown to improve the tissue microenvironment. For example, Fu et al. [123] demonstrated that MSC-Exos can remodel glutamine and ammonia metabolism in hepatocytes by modulating glutamine synthetase activity, helping to enhance the microenvironment of the injured liver and alleviate liver fibrosis.

## 9. Conclusions and Future Perspectives

MSCs are a type of stem cell with low immunogenicity, self-renewal capacity, and multi-directional differentiation potential. MSC-based therapies exhibit considerable potential in regenerative medicine and clinical applications. However, MSC-based therapies still face certain challenges in clinical applications, including potential tumorigenic risks and insufficient targeting efficiency. Exos, as key mediators of MSC functions, possess properties similar to MSCs and have attracted significant research interest. Exos play crucial roles in immune responses, facilitate intercellular communication, and offer potential in treating various diseases. Compared to MSC-based therapies, MSC-Exos offer a cell-free alternative that reduces the risks associated with cell therapy and minimizes the likelihood of clearance by the host immune system. Current evidence indicates that MSC-Exos play a crucial role in treating AIDs, significantly improving symptoms and enhancing patient prognosis.

AIH is an autoimmune disease marked by an imbalance in immune responses, leading to the apoptosis of regulatory T cells and excessive antibody production by B cells. Currently, effective treatments for AIH remain limited, and the disease is prone to progressing to liver fibrosis and cirrhosis in its advanced stages. Exos, as a type of extracellular vesicle, can influence immune cells by carrying and delivering various small molecules (such as DNA, RNA, and proteins). They modulate intracellular signaling pathways and cytokine secretion, thereby inhibiting B lymphocyte activity, reducing antibody production, and promoting the homeostasis of T and B cells. Additionally, MSC-Exos can also facilitate the differentiation of macrophages into anti-inflammatory macrophages and modulate the function and activity of DC and NK cells. MSC-Exos can also enhance therapeutic efficacy by carrying therapeutic agents, such as DEX. Additionally, after genetic and chemical modifications, MSC-Exos exhibit improved targeting of damaged tissues, thereby increasing treatment efficiency. Furthermore, MSC-Exos can ameliorate liver fibrosis by modulating HSCs, and they promote liver cell regeneration by delivering growth factors and reducing oxidative stress, which helps inhibit hepatocyte apoptosis.

Although MSC-Exos offer promise for the treatment of AIH patients, the following problems still exist: (1) The need for more standardized methods for the extraction, preparation, and storage of MSC-Exos: Currently, the extraction of MSC-Exo remains inefficient, and existing purification methods are inadequate. To address these issues, it is essential to develop and standardize operating procedures and quality control standards for cell culture, MSC-Exo extraction, and purification processes. Additionally, due to the unstable nature of MSC-Exos, further research is needed to optimize storage conditions and ensure the preservation of their therapeutic efficacy. (2) Delivery route and dosage: Research is ongoing to identify the most effective methods for delivering MSC-Exos and determining the optimal dosage. Different administration routes (e.g., intravenous, local injection) can significantly impact therapeutic efficacy. Additionally, comprehensive in vivo studies are required to establish the optimal dose of MSC-Exos for maximal therapeutic benefit. (3) Safety: While MSC-Exos generally exhibit high safety and low immunogenicity compared to MSC cell therapy, there remains a potential risk of adverse reactions and immune responses, particularly with prolonged use or high dosages. (4) Regulatory and approval challenges: In numerous countries, MSC-Exo therapy has not yet received official approval or clear regulatory guidelines, limiting its widespread clinical application. In addition, due to insufficient preclinical and clinical studies, further exploration in this domain is imperative. In conclusion, while MSC-Exos hold promise as a novel strategy for AIH treatment, further in-depth research and theoretical validation are essential to enable their practical application in clinical settings.

## Figures and Tables

**Table 1:** Therapeutic effects and relevant mechanism of MSC-Exos/EVs in AIH.

Type	Source	Disease	Target Cell	Efficacy	Mechanism	Ref.
MSC-Exo	BM	hepatic S100-induced AIH	hepatocytes	Reversed liver injury and reduced hepatocyte apoptosis	Regulated NLRP3 and caspase-1 by miR-223	[54]
MSC-Exo^miR-223-3p^	BM	hepatic S100/CFA-induced AIH	macrophage	Decreased macrophage release of cytokines IL-1β and IL-6	Reduced expression of genes STAT3 and p-STAT3	[55]
MSC-Exo@DEX	BM	Con A-induced AIH	hepatocytes, macrophage	Reducing inflammation to protect liver tissue integrity	Decreased secretion of TNF-α, IFN-γ and IL-1β	[56]
MSC-CM	BM	Con A-induced AIH	hepatocytes	Reduced liver enzymes and inflammatory cytokines	Reducing the amount of activated TCD4 and TCD8	[57]
MSC-EV	UC	Con A-induced AIH	T cells	Decreased IFN-γ and reduced CD4 T-cell activation	Metabolically reprogram CD4 T cells	[58]

**Table 2:** The MSC Therapy and MSC-Exo Therapy related clinical research in AIH and late complications.

NCT Number	Study Title	Conditions	Interventions
NCT02997878	Selected Mesenchymal Stromal Cells to Reduce Inflammation in Patients with PSC and AIH	Cholangitis, Sclerosing/Hepatitis, Autoimmune	MSCs
NCT01661842	Umbilical Cord Mesenchymal Stem Cells for Patients with Autoimmune Hepatitis	Autoimmune Hepatitis	UC-MSCs
NCT06629909	Safety and Feasibility of Human Umbilical Cord Mesenchymal Stem Cell-Derived Secretome in the Treatment of Liver Cirrhosis: a Comprehensive Evaluation of Fibrosis Reduction, Immunomodulation, and Hepatic Regeneration: a Single Center, Randomized, Phase I Clinical Trial	Liver Cirrhosis	UC-MSCs secretome
NCT05940610	The Safety and Efficacy of MSC-EVs in Acute/Acute-on-Chronic Liver Failure	Acute-On-Chronic Liver Failure/Acute Liver Failure	MSC-EVs
NCT05881668	MSC-EV in Acute-on-Chronic Liver Failure After Liver Transplantation	Liver Failure, Acute on Chronic	MSC-EVs
NCT05871463	Effect of Mesenchymal Stem Cells-derived Exosomes in Decompensated Liver Cirrhosis	Decompensated Liver Cirrhosis	MSC-Exos

## Data Availability

All data are incorporated into the article.
